# Clay-Based Nanocomposite Hydrogels for Biomedical Applications: A Review

**DOI:** 10.3390/nano12193308

**Published:** 2022-09-23

**Authors:** Cezar Tipa, Maria T. Cidade, João P. Borges, Luis C. Costa, Jorge C. Silva, Paula I. P. Soares

**Affiliations:** 1 CENIMAT|i3N, Department of Materials Science, School of Science and Technology, NOVA University Lisbon, 2829-516 Caparica, Portugal; 2 I3N and Physics Department, University of Aveiro, 3810-193 Aveiro, Portugal; 3 CENIMAT|i3N, Department of Physics, School of Science and Technology, NOVA University Lisbon, 2829-516 Caparica, Portugal

**Keywords:** biomedical applications, clay nanoparticles, hydrogel, nanocomposite

## Abstract

In recent decades, new and improved materials have been developed with a significant interest in three-dimensional (3D) scaffolds that can cope with the diverse needs of the expanding biomedical field and promote the required biological response in multiple applications. Due to their biocompatibility, ability to encapsulate and deliver drugs, and capacity to mimic the extracellular matrix (ECM), typical hydrogels have been extensively investigated in the biomedical and biotechnological fields. The major limitations of hydrogels include poor mechanical integrity and limited cell interaction, restricting their broad applicability. To overcome these limitations, an emerging approach, aimed at the generation of hybrid materials with synergistic effects, is focused on incorporating nanoparticles (NPs) within polymeric gels to achieve nanocomposites with tailored functionality and improved properties. This review focuses on the unique contributions of clay nanoparticles, regarding the recent developments of clay-based nanocomposite hydrogels, with an emphasis on biomedical applications.

## 1. Introduction

Current technological requirements for new biomedical advances include new or improved materials with adequate functionality and performance. Materials with unique properties for applications in medicine have emerged due to the continuous evolution and key discoveries in the biomedical sciences and biotechnological fields. This growth has a significant impact on the ongoing development of biomaterial technology and the current practice of medicine. Over recent decades, the integration of nanotechnology with other fields of science has captivated a great deal of attention, resulting in numerous approaches toward the manufacturing of improved materials and physiologically relevant systems [1].

Nanomedicine is still a developing and demanding medical field, and it has produced diverse nanomaterials and biological devices such as optical tools, biosensors, imaging probes, advanced drug delivery systems, and vehicles for proteins and enzymes, among many others [2]. Combining different and wisely chosen materials, a synergistic effect emerges in which composite scaffolds present advantageous properties compared to the sum of their parts while reducing or eliminating their limitations. Developments in nanobiotechnology, material design, and surface functionalization techniques have also enabled the production of functional biomaterials that can mimic the inherent attributes of the extracellular matrix (ECM), a quality which can be necessary in several biomedical applications [3].

The ECM is a tridimensional (3D) noncellular viscoelastic and dynamic nanofibrous network. The main function of the ECM is to regulate cell proliferation, differentiation, and migration, and to provide a physical and biochemical foundation for cells. Consequently, developing biomaterials to mimic the ECM should include precise control over their physical, chemical, and biological properties, details which are critical for the growth of functional tissues [3]. Precisely for their common characteristics with living tissues and their structural resemblance to in vivo ECM, among other things, hydrogels have been extensively studied.

### 1.1. Hydrogels

It is worth noting that among the varied techniques for the production of scaffolds, the design and fabrication of hydrogels has manifested appealing characteristics and attained increased popularity among researchers around the globe, amplifying the pace of investigation for hydrogels in nanomedicine [2].

**Figure 1:**
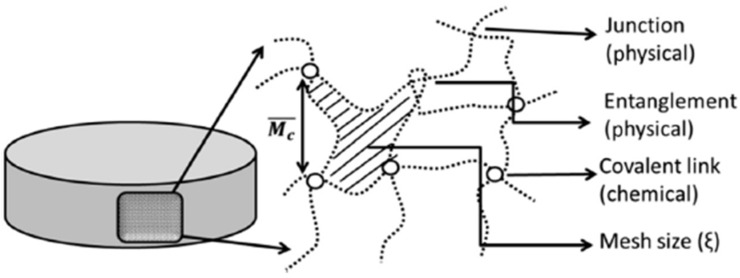
Microstructural parameters of hydrogels. (Reprinted with permission from [5]. Copyright 2020, Elsevier).

Hydrogels are entangled networks of, usually, natural or synthetic polymeric chains interconnected by crosslinking points, producing a 3D structure (Figure 1). This 3D structure can absorb and retain significant amounts of aqueous fluids due to its hydrophilic nature [4]. 

Natural polymers include collagen, hyaluronic acid (HA), alginate, agarose, gelatin, gellan gum (GG), and chitosan, among others. These polymers are usually biocompatible but can be difficult to process. Alternatively, synthetic polymers are materials with controllable and well-replicated chemical and physical properties. Some examples include poly(ethylene glycol) (PEG) and poly(acrylic acid) (PAA) derivatives [6]. However, non-biocompatible materials and solvents used during the production process as well as cytotoxic byproducts, among other harmful residues, are a real concern. Semi-synthetic polymers such as gelatin methacrylate (GelMA) and hydroxypropyl methylcellulose (HPMC) are chemical modifications of natural polymers which combine both the benefits of biopolymers and the reproducible characteristics and chemical control attributed to synthetic polymers. Hydrogels’ crosslinking can either be of chemical or physical nature. Chemical gels are produced by irreversible covalent bonds, producing a 3D network that swells without dissolving. Their preparation can include polymerization in the presence of a cross-linker, or covalent cross-linking of a certain polymer with diverse procedures, resulting in strong non-reversible bonds [7]. Physical gels are reversible and amorphous networks of hydrophilic polymers that can dissolve in aqueous solutions at different rates. These networks are maintained by weak non-covalent bonds, such as hydrogen bonds, hydrophobic interactions, and Van der Waals forces [8].

**Figure 2:**
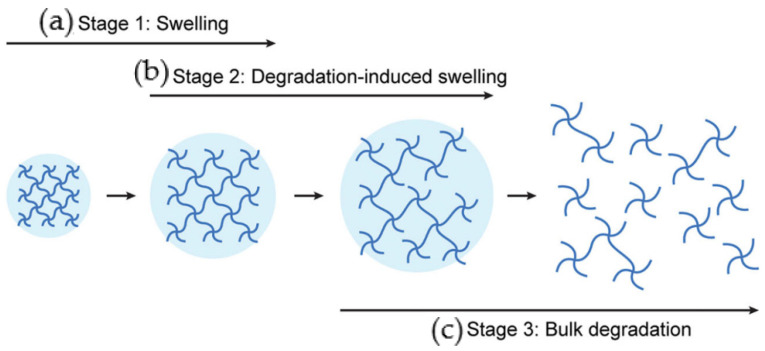
Schematic representation of the temporal changes in degradable hydrogels’ shape following implantation: (**a**) the first stage includes swelling driven by the osmotic pressure; (**b**) during the second stage, the continuous swelling of the polymer network induces its degradation; (**c**) the last stage includes bulk degradation, leading to complete disintegration of the polymeric network. (Reprinted with permission from [11]. Copyright 2015, John Wiley and Sons).

These biomaterials are studied mainly due to their swelling, highly hydrated and soft porous structure, high biocompatibility, function as drug protectors designed to hold or release materials, and biodegradability (Figure 2) [9]. Additionally, hydrogels can be prepared using polymers with specific functional groups on their backbone, thus producing a “smart hydrogel” that responds to different environments and external stimuli. This response can cause physical and/or chemical structural changes in the hydrogel structure [8]. Hydrogels have intrinsic properties that are ideal for scaffolds: flexibility and soft structure. Consequently, when in contact with tissues, the hydrogel causes minimal harm while providing the needed permeability to water and nutrients, sustaining cell growth [10].

Several hydrogels have already been used in the past decade for various biomedical applications that require cooperative exchange with dynamic cellular microenvironments. These hydrogels possess structural similarities to the ECM found in vivo by mimicking native tissue microenvironment with similar properties common to living tissues. For example, the low interfacial tension between the hydrogel surface and biological fluids minimizes the occurrence of an immune reaction by better matching the living tissue properties [12,13].

In cases where the dimensions of the injured tissues are too intricate to be replicated or a given defect site is not accessible, injectable hydrogels are an alternative. Hydrogels can adapt to environmental conditions due to their flexible nature while maintaining their unique properties. Therefore, a solution with subsequently triggered network formation can be used as an injectable hydrogel system [12]. Thus, damaged tissues can be efficiently filled in a convenient and minimally invasive procedure to aid regeneration, circumventing the need for complex surgeries and minimizing the damage to the surrounding healthy tissue [14].

Hydrogels with previously unattainable functions and behaviors have been developed due to the evolving innovations from biomolecular engineering and polymer chemistry [15]. However, their broad applicability in the biomedical industry is a significant limitation due to poor mechanical strength, lack of structural support, absence of cell-binding sites, and inadequate degradation behavior [16]. As a result, researchers have explored the inclusion of a vast range of nanoparticles (NPs) within hydrogel networks, enabling the design of materials with enhanced physiological performance and tailored functionality. This combination produces nanocomposites or hybrid hydrogels. Besides the polymers already discussed, other bio-based materials can also form hydrogels, which have yet to be explored in combination with inorganic reinforcements/fillers. Some examples include animal-derived proteins (e.g., elastin, silk) and plant proteins (e.g., cellulose, soy protein, zein) [3].

### 1.2. Clay Nanoparticles

It is well known that the smaller the reinforcement, the greater the prospects of obtaining well-developed materials for structural improvement and overall functional benefits [17]. The growing interest in layered silicates for biomedical applications [18] is due to their distinctive physicochemical properties [19], such as crystalline structure, colloidal particle size and shape, layer charge density, specific surface area, cation exchange, and swelling capacity [20]. Also, in addition to being an abundant and low-cost material, the amount of clay nanoparticles (dispersed phase) required for incorporation into a polymeric matrix (continuous phase) in order to convey significant variations is very low [21]. For clay-reinforced hydrogels and assigned applications, benefits can be in the form of improved mechanical and load-bearing capacity, gel swelling stability, tuning rheological behaviors, adding-up bio-adhesion and cell interactions, and improving the 3D processability of bioinks and the shape fidelity of 3D-printed structures, among others [22]. These properties vary based on the clay composition and crystal structure.

**Figure 3:**
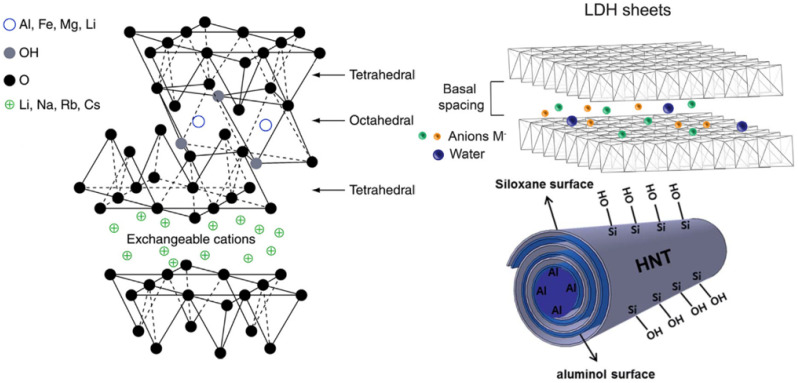
Structure of the most-used clay minerals. Cationic 2:1 layered silicates (such as Smectites). (Reprinted with permission from [25]. Copyright 2017, Elsevier). Anionic layered double hydroxides (LDH). (Reprinted with permission from [26]. Copyright 2021, Elsevier). Basic 1:1 kaolinite silicate rolled over to form halloysite nanotubes (HNTs). (Reprinted with permission from [27]. Copyright 2017, Royal Society of Chemistry).

**Figure 4:**
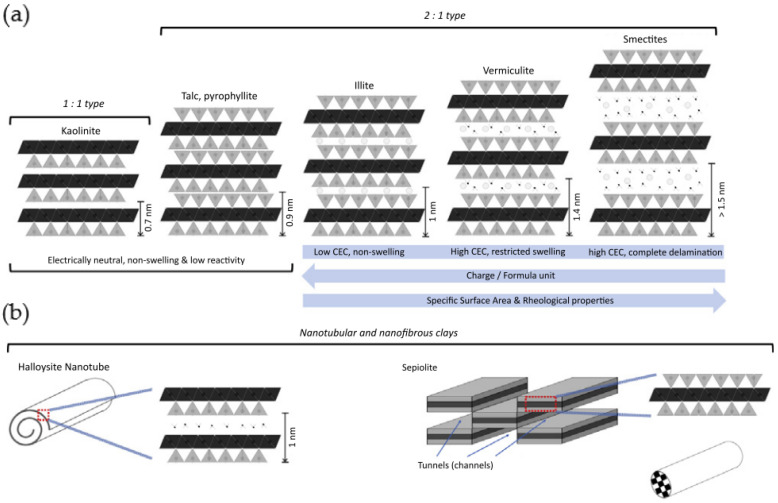
(**a**) The structure and reactivity of different groups of clay minerals. The reactivity of clays depends on their swelling capacity. Typically, these clay layers (aluminosilicate layers or 2:1 phyllosilicates) have an overall negative surface charge, due to isomorphic substitutions in the O (Al^3+^ with Fe^2+^ or Mg^2+^) sheets and T (Si^4+^ with Al^3+^) sheets, which is balanced out by exchangeable metal cations such as Fe^2+^, Ca^2+^, Na^+^, Li^+^, and Mg^2+^, occupying the clay interlayer space, as well as water molecules. The capacity for hydration, swelling and dispersion increases with the negative charge value. Thus, between the negatively charged surfaces of the clay layers a positively charged layer is formed, shaping the whole crystal structure when stacked up. The aspect ratio of clay layers (length/thickness ratio) is very high (>1000), since each layer is around 1 nm thick and 300 Å to several microns wide in lateral dimensions. (**b**) Distinctive structue from other clay minerals: halloysites (from the 1:1 family) and sepiolites (from the 2:1 family). (Reprinted with permission from [29]. Copyright 2018, Elsevier).

Clay minerals can be classified according to the charge of their layers into neutral (nonionic), cationic, and anionic clays (Figure 3). The silicon, metal, oxygen, and hydroxyl groups of clays are organized in 2D structures named sheets, where the clay mineral structure is composed of two principal units: tetrahedral silica (T) and octahedral alumina (O) sheets. Tetrahedral sheets (T) contain silicon surrounded by four oxygen atoms, while the octahedral sheets (O) are composed of a metal like magnesium and aluminum, surrounded by eight oxygen atoms. These layer-by-layer structures are classified into different types (mainly 1:1 or 2:1) depending on the ratio and layering of silica (T) to alumina (O) sheets (Figure 4a) [23]. Anionic layered double hydroxides (LDH) possess stacked layers with a brucite-like structure similar to hydrotalcite, that holds exchangeable anions in their interlaminar space (as well as water molecules) [24]. The 1:1 (or T-O layers) single two-sheet mineral clays are held/linked together by hydrogen bonding between the hydroxyl group in octahedral sheets and oxygen in tetrahedral sheets. Their interlaminar space is occupied by water molecules, e.g., kaolinite, perlite, halloysite, serpentine, etc. The 2:1 (or T-O-T layers) three-sheet mineral clays are composed of a single O sheet between two T sheets. The stacking of these T-O-T layers originates a Van der Waals gap between layers of clays such as montmorillonite (MMT), laponite, saponite, or hectorite, among others.

Since no isomorphous substitution occurs in the 1:1 clay mineral group, they are electrically neutral and have weak hydrogen bonds and Van der Waals forces, no structural (permanent) charges, generally low cation exchange capacity (CEC), and less biomedical interest when compared to the 2:1 type due to their inability to undergo interlayer swelling in water. However, halloysite (HNT) is an exception, since its hydrated 1:1 kaolinite sheets roll up several times into tubular clay nanomaterials (inside O sheet and outside surface T sheet), enabling attractive features for biomedical applications such as total pore volume and high specific surface area [18].

The basic 2:1 (T-O-T) structure without any substitution of atoms is called pyrophyllite, and its layers do naot swell in water. As with kaolinite (of the 1:1 clay family), pyrophyllite and talc are of low reactivity, and their layered structure is electrically neutral (consequently non-swelling and of low reactivity). Contrarily, for smectite and vermiculites, the occurrence of isomorphous cation substitutions leads to clay particles with a different net charge, surface reactivity, ion exchange capacity, gelation, and swelling behaviors. The smectite group is mainly known for montmorillonite (MMT) and laponite (a synthetic hectorite) which are the most investigated among all phyllosilicates for biomedical applications due to their ability to show extensive interlayer expansion/swelling. In addition, their relatively low net charge enables complete delamination caused by osmotic swelling, thus exposing a chemical-rich surface. These structural changes allow for the interaction with various biomolecules or polymers in addition to conferring unique rheological properties once dispersed in water. Contrarily, vermiculites and ilites display a higher layer charge that limits water accessibility to the interlayer region, which inhibits delamination due to restricted swelling, gelling tendency, and overall reactivity. However, they still possess high specific surface area (SSA) and cation exchange capacity (CEC). Lastly, as halloysites (from the 1:1 family), sepiolites are distinctive from other clay minerals (in the 2:1 family) due to their unique inverted 2:1 structure, which offers these clay minerals porosity and fibrous morphology with high SSA (Figure 4b) [28]. 

Besides their use as reinforcement agents, clay nanoparticles have also been used in the development of hybrid materials for specific medical applications such as regenerative medicine and as vehicles to efficiently deliver therapeutic molecules [30]. 

The ability of clay minerals to bind therapeutic agents has been known for more than 50 years, when clinicians noted the unusually reduced existence of drugs in the blood stream (reduced systemic bioavailability) when patients were simultaneously receiving clay-based antidiarrheal treatments [29,31]. This feature and binding mechanism of clay minerals has been extensively studied and used to carefully control the release of several pharmaceutical drugs [32] for several different pharmaceutical applications, such as skin healing [33], among others [34]. Clay association mechanisms with biomolecules include (i) intercalation within clay’s interlaminar space through cation exchange reaction, (ii) adsorption on the layer surfaces of clays via electrostatic interactions, (iii) binding of polar biomolecules at hydrophilic and hydrophobic sites, among other interactions such as (iv) hydrogen bonding, Van der Waals interactions, ligand exchange, and cation/water bridging [22].

Several types of clay minerals such as halloysite nanotubes [35], montmorillonite [36], laponite [37], and layered double hydroxide (LDH) [38], among others, have been studied extensively (i) for their ability to interact with biomedical scaffolds, (ii) as transport vehicles for advanced drug delivery, (iii) due to improved interactions with cells and tissues, and (iv) for several potential biomedical applications [24].

The incorporation of clay nanoparticles has also been used to control rheological behaviors and shear thinning characteristics of available bioinks in order to optimize the processability and 3D printability of complex structures [29]. There are also studies where clays are applied to aid in the formation of new blood vessels (by binding of the vascular endothelial growth factor) [33] and to stimulate bone formation at reduced doses of an osteoinductive growth factor (bone morphogenetic protein) [39]. 

### 1.3. Clay-Based Nanocomposite Hydrogels

The development of nanomaterials appears to be, based on recent research, the future of evolving nanomedicine technology [26]. In 2002, Haraguchi [2] produced the first documented clay-based nanocomposite hydrogel, which is now considered a turning point in this research field [15]. The selection of the clay nanoparticle and hydrogel type, followed by the preparation and crosslinking method, enables the control over the intermolecular interactions between nanoparticles and hydrogel [40].

However, further research was required to determine what makes biological tissues so complex and why their performance surpasses the engineered polymeric materials used in biomedical applications [41]. While studying the characteristics of certain biological tissues, it was noticed that a polymer nanocomposite structure with a softer biopolymer matrix is often responsible for their mechanical properties [42]. The ECM offers not only a 3D environment for cells to grow but is also mechanically stable with sufficient space to facilitate vascularization and transport of nutrients and cells. Given the complexity of ECM functions, this inspired researchers to design and engineer nanocomposite materials with unique and synergistic combinations of suitable hard and soft components, enabling the design of a broad spectrum of optimal functionalities [43].

Even if nanotechnology advances allow for the generation of diverse, well-defined nanomaterials (in terms of properties, sizes, and shapes), their use in biomedical fields remains an ongoing challenge due to the need for convenient sterilization, ease in processing procedures, and application safety of all materials included, as well as interface compatibility between nanomaterials and polymeric matrices [3].

To address the current limitations and need for safe and multifunctional systems, researchers have been focusing on designing physiologically friendly hydrogels reinforced with biocompatible clay nanoparticles [44]. The incorporation of nanoparticles into hydrogel’s network/structure/matrix can occur by absorption or homogeneous dispersion but also works as nanoreactors for their preparation [15]. Nanocomposite hydrogels can be defined as reinforced 3D networks (i) of crosslinked polymeric matrices and reinforcing nanofillers or (ii) network/matrix simultaneously formed in the presence of nanoparticles either by physical or chemical crosslinking [45]. In chemical crosslinked hydrogels, polymer chains can be strongly adsorbed to the surfaces of different clay nanoparticles and provide enough attachments to constitute a strong 3D network structure [46].

**Figure 5:**
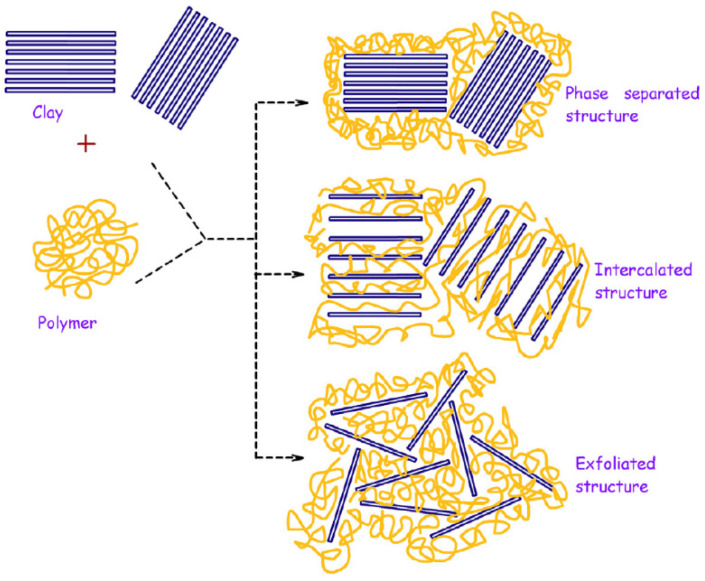
Schematic representation of composites produced from the interaction of layered silicates and polymeric networks. (Reprinted with permission from [48]. Copyright 2017, Elsevier).

Combining two different materials can offer an array of possible interactions and interface conditions, resulting in new structural properties and overall behavior [2,47]. For example, the interaction of polymeric networks with layered silicate clays is described in the literature (Figure 5) mostly by these nanocomposite structures: (i) conventional composite (2 separated phases), (ii) intercalated nanocomposite (polymer intercalated in between the lamellar structure), and (iii) exfoliated nanocomposite (lamellae homogeneously dispersed in the polymer matrix) [23,31].

**Figure 6:**
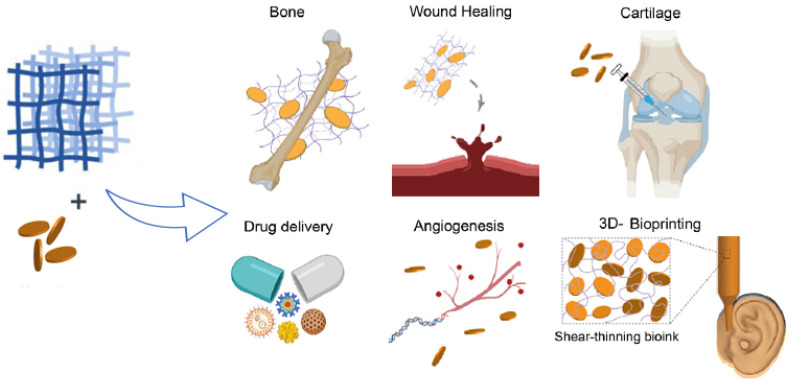
Schematic representation showing the broad applicability of clay-based nanocomposite hydrogels in the biomedical industry. (Adapted with permission from [26]. Copyright 2021, Elsevier).

The synergistic combination of polymeric matrices, therapeutic compounds, and nanometric clay fillers meets various requirements for different applications, especially in biomedicine [10]. This review describes the most recent state of the art concerning the fabrication of clay-based nanocomposite hydrogels for biomedical applications such as tissue engineering, bone and cartilage repair, drug delivery, wound healing, bioprinting as well as biosensors, cell culture, stem cell bioengineering, and antimicrobial applications (Figure 6). The materials used for their fabrication as well as their properties and ongoing developments will also be addressed.

## 2. Nanocomposites Features and Characterization

The novelties behind clay nanocomposite hydrogels consist of new approaches to synergistically synthesize, modify, and reinforce conventional polymeric matrices using clay nanofillers and the possible benefits that spring out of it for biomedical applications. This section addresses the main techniques used to produce nanocomposite hydrogels with clay nanofillers and delves deeper into the main properties that are impacted by this interaction.

### 2.1. Synthesis

**Figure 7:**
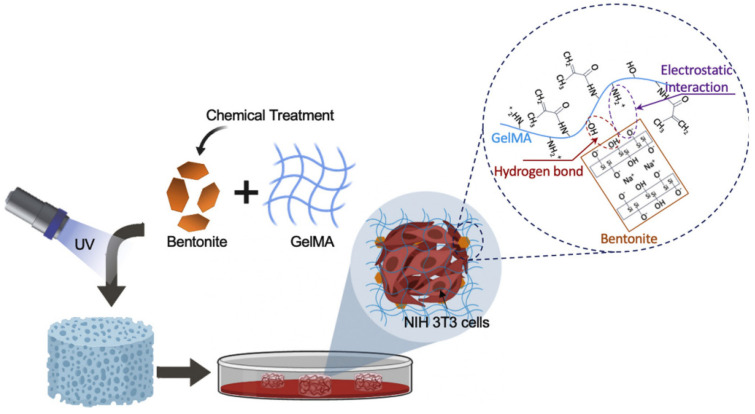
Schematic representation of bentonite-GelMA fabrication with NIH 3T3 fibroblast cells. (Reprinted with permission from [49]. Copyright 2020, Elsevier).

**Figure 8:**
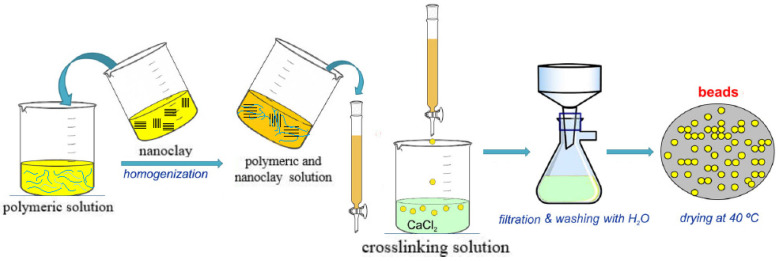
Schematic representation of Ca^2+^ alginate/clay nanocomposite hydrogel beads synthesis procedure by [50]. (Adapted with permission from [34]. Copyright 2019, Springer Nature).

Depending on the desired characteristics of polymer clay nanocomposite hydrogels, different techniques can be applied in their preparation. For example, Sakr et al. [49] developed a nanocomposite hydrogel based on bentonite (Bent) clay NPs incorporated into a polymeric matrix of gelatin methacryloyl (GelMA) (Figure 7). The GelMA synthesis was a 7-day fabrication process. Bentonite NPs were mixed with a GelMA prepolymer solution and sonicated for 30 min to avoid agglomerations and aggregations. A (2-hydroxy-4′-(2-hydroxyethoxy)-2-methylpropiophenone was used as a photo-initiator to produce free radicals, thus initiating the polymerization simultaneously with the formation of an irreversible cross-linked 3D hydrogel. The final prepolymer solution was mixed with NIH 3 T3 fibroblast cells and irradiated with UV light for 60 s to produce the cell–bentonite–GelMA composite hydrogels. For potential drug delivery applications, Silva Fernandes et al. [50] used another synthesis route and prepared Ca^2+^ alginate/clay nanocomposite hydrogels from an optimized bead forming procedure. The zeolite and Cloisite-Na^+^ nanoclay sonicated dispersions were added to the sodium alginate solution. A 2% solution (*w*/*v*) was extruded using a syringe pump operating at a rate of 30 mL/h into goblets containing distinct concentrations of calcium chloride. The drops, when in contact with calcium chloride solutions, immediately turned into beads that were kept immersed for 2 h to ensure complete crosslinking. The nanocomposite beads were finally washed to remove the excess calcium and dried (Figure 8).

Similarly, to load and release therapeutic agents, Boruah et al. [51] designed a biocompatible hydrogel based on carboxymethylcellulose-g-polyacrylic acid (CMC-g-PAA) and organo-montmorillonite clay to produce a nanocomposite system for vitamin B12 release. The nanocomposite system was synthesized by radical graft polymerization, potassium persulfate (KPS) being used as initiator, and crosslinked with methylene bis-acrylamide (MBA). Vitamin B12 was loaded by placing the hydrogel in the drug solution as long as necessary for complete encapsulation. The vitamin B12-loaded swollen hydrogel was then kept in an oven at 30 °C until complete drying was achieved. Nanocomposite films using a polyvinyl alcohol (PVA) hydrogel and Na-montmorillonite were prepared by Kokabi et al. [52] for wound management and care. Differently from the studies discussed before, the original clay was modified by cation exchange reaction using cetyl trimethyl ammonium bromide (CTAB), giving rise to organophilic montmorillonite. To prepare the nanocomposite hydrogel, PVA and clay were dissolved in water and mixed. Following this, the mixture was poured into plastic molds and submitted to three freeze–thaw cycles to obtain the final nanocomposite.

**Figure 9:**
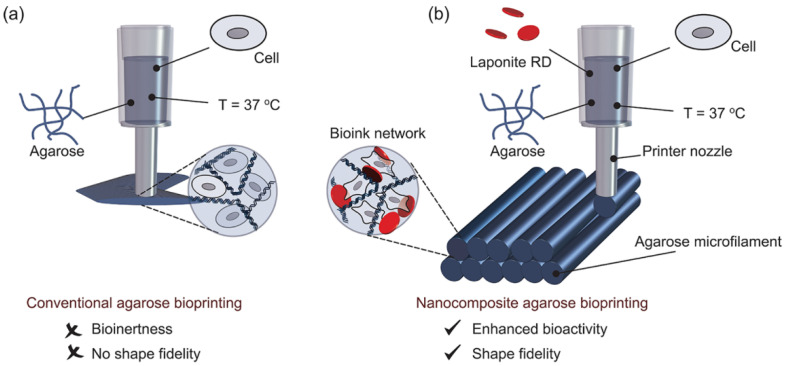
Schematic representation of: (**a**) conventional bioprinting (without a nanofiller); and (**b**) nanocomposite bioprinting, including polymeric bioinks with a nanofiller (laponite clay). (Reprinted with permission from [55]. Copyright 2019, American Chemical Society).

Additive manufacturing strategies are currently widely exploited for tissue and organ regeneration. One of the most-investigated additive manufacturing techniques is extrusion-based 3D bioprinting. This technique uses hydrogel bioinks that can be tailored to achieve the specific requirements of 3D bioprinting [53]. To print stable and complex 3D structures from hydrogel-based bioinks, a few criteria must be satisfied: (i) the bionk precursor solution must be extrudable/printable, (ii) an optimal gelation time is essential for the extruded bionk to self-support in layer by layer constructs, (iii) the layer-by-layer stacking has to be firmly bound to prevent delamination; (iv) the final cross-linked 3D printed scaffolds should be physiologically stable in aqueous conditions (without critical swelling) and be able to support load bearing biological applications. Therefore, hydrogel bioinks selection should consider their biocompatibility, rheological behavior and structural stability throughout the process [54]. Furthermore, the use of nanofillers can significantly change the physiochemical properties of hydrogels, thus enabling their use in 3D bioprinting (Figure 9) [55].

This use of nanofillers for improved bioink manufacturing was demonstrated in a recent work, wherein Hu et al. 2021 [56] produced a novel bioink for 3D printing composed of thermoresponsive block copolymer poly(2-methyl-2-oxazoline)-b-poly(2-n-propyl-2-oxazine) (PMeOx-b-PnPrOzi) and nanoclay laponite. The nanocomposite developed was loaded into a printing cartridge while still in liquid state directly after being withdrawn from the refrigerator where it was stored at 7 °C. It was then set up in the 3D printer and driven pneumatically through the nozzle at 600 mm·min^−1^, to print the hydrogel onto polystyrene plates at room temperature and obtain 3D-printed structures.

### 2.2. Swelling

Several polymers interact physically with clay NPs, thus significantly affecting their swelling capacity. The ability to retain specific molecules, combined with the stimulus-responsive swelling, water expulsion, absorption and content release, makes hydrogels a likely candidate for the delivery of therapeutic agents [43].

**Figure 10:**
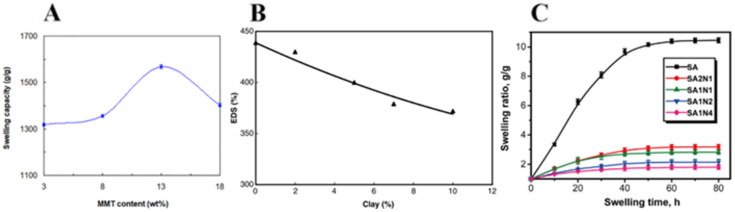
Nanocomposites’ swelling variation with clay content. (**A**) Swelling capacity of CS-g-poly(AA-co-AAm)/PVP/MMT samples. (Adapted with permission from [58]. Copyright 2019, Advanced Pharmaceutical Bulletin). (**B**) Swelling degree of PVA–MMT hydrogels. (Adapted with permission from [52]. Copyright 2017, Elsevier). (**C**) Swelling ratio of sodium alginate SA and SA/HNTs hydrogels. (Adapted with permission from [60]. Copyright 2017, Elsevier).

To improve the dissolution patterns of poly(ethylene oxide) (PEO) films, Gaharwar et al. [57] developed laponite cross-linked PEO nanocomposite films and found that the addition of silicates reduces the hydration degree as well as network porosity. It also improved the structural stability of the films when submersed in PBS, whereas pure PEO films readily dissolve in within minutes. They theorized that the silicates’ ionic interaction with the polymeric network and the water molecules dictated the structural stability of the nanocomposite films immersed in PBS. In a more recent work, Panahi et al. [58] evaluated the effect of MMT loading on the water absorption capacity of CS-g-poly(AA-co-AAm)/PVP/MMT hydrogels. As shown in Figure 10A, MMT content increased from 3 wt.% to 13 wt.%, boosting the swelling capacity of the hydrogel up to 1568 g/g. This can be considered an unusual and very interesting behavior, since most reports attach a diminished swelling capacity to the influence of clays. However, a considerable reduction occurs with MMT beyond 13 wt.%. They stated that for MMT loadings lower than 13 wt.%, a higher expansion in the hydrogel network is a consequence of the difference in osmotic pressure between swelling medium and hydrogel samples. Under these circumstances, the enlarged available holes within the hydrogel matrix can absorb more water molecules, increasing the swelling capacity. Excessive reinforcement of the physical crosslinking density with further MMT content increases up to and beyond 18 wt.% decreases the water absorption capacity. Similar swelling increases to the profile of starch-grafted polyacrylic acid-based hydrogel was observed by Datta Chaudhuri et al. [59] with the addition of bentonite clay. Since this important characteristic relates to the composite films’ ability to absorb wound fluids and exudates, Kokabi et al. [52] also measured the swelling characteristics of PVA-clay film samples. As expected, Figure 10B reveals a decrease in the swelling ability of PVA hydrogels when clay particles are incorporated. Even so, they concluded that the resultant swelling capacity was still suitable for the intended applications. Regarding the swelling capacity, analogous findings were observed in most studies, such as the following examples. Huang et al. [60] explored the effect of halloysite nanotubes (HNTs) on the swelling abilities of sodium alginate (SA) composite hydrogels (Figure 10C). The incorporation of HNTs into SA hydrogels, gradually decreased the swelling ratio with higher loadings. For SA1N4, the swelling ratio after 80 h is 1.8 g/g, in contrast with the 10.5 g/g for pure SA. The incorporation of HNT in SA hydrogel decreases the polymeric network’s hydrophilicity, consequently decreasing the number of interactions with water, thus decreasing the water absorption ratio of the nanocomposite hydrogel. Boruah et al. [51] developed CMC-g-polyacrylic acid/OMMT nanocomposite hydrogels and realized that the swelling capacity of the nanocomposite hydrogels with the addition of clay nanoparticles followed a similar trend to the influence of crosslinker content, i.e., swelling (water absorbency) diminished with the increase in clay content (since clay minerals can act as additional networks reinforcements). 

### 2.3. Rheological Behavior

Injectable hydrogels require a precursor solution that can be easily injected and that can gelify in situ, thus producing a mechanically adhesive and robust hydrogel. These injectable hydrogels are very interesting for minimally invasive therapies from a clinical perspective. Injectability, flow behavior, and gelation temperature, among other characteristics of nanocomposite hydrogels, can be determined by rheological characterization.

Huang et al. [61] designed PEG hydrogels reinforced by functionalized LDH nano-crosslinkers and witnessed a dramatic increase in the storage modulus from about 20 to 840 Pa when the PD-LDH content increased from 0 to 1.33 wt.% in a 2 wt.% polymeric phase. This indicates an evident impact on the viscoelastic properties and overall integrity of hydrogels with clay addition. In terms of gelation time parameters, Su et al. [62] enhanced the gelation of injectable silk fibroin hydrogels with laponite. The gelation of regenerated silk fibroin (RSF) aqueous solution requires a few weeks at 4 °C, about 10 days at 37 °C and only about 40 min at 95 °C. Laponite reinforcement shortened the gelation time of the RSF solution (at 37 °C) from 10 days to less than 3 h when incorporated with small amounts of LAP (0–5%, *w*/*w*). Similarly to the work discussed before, this nanocomposite hydrogel’s storage modulus (G’) increased from about 80 kPa to 200 kPa when the laponite content increased from 1% to 5% while that of the pristine RSF hydrogel is only 30 kPa. 

**Figure 11:**
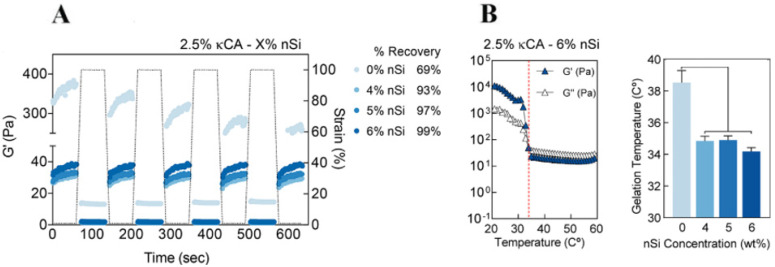
Rheological changes to the hydrogel with clay addition. (**A**) Percent recovery of kCA−nSi bioink at 37 °C with the introduction of nSi. (**B**) Temperature ramp (60 to 20 °C) to display the gelation of bioink compositions. (Adapted with permission from [63]. Copyright 2017, American Chemical Society).

A bioink formulation should efficiently deform and reform several times during the printing process while retaining flow behavior without consequences to its integrity. Thus, rheological characterization is extremely valuable in evaluating the viscoelastic and responsive properties of developed bioinks. Wilson et al. [63] designed a thermo-responsive nanoengineered bioink for 3D printing. Pure kappa-carrageenan (κCA) cannot be printed due to poor percent recovery and 40 °C gelation temperature. Therefore, the effect of nSi addition on recovery and gelation temperatures was investigated. A high percent recovery (above 90%) of the storage modulus and shorter recovery time (<5 s) of the network properties were achieved for all the clay-reinforced bioinks tested ((Figure 11A). Additionally, all bioinks reinforced with clay minerals exhibited considerably lower gelation temperatures (around 35 °C) (Figure 11B), which allowed for the printing of 3D structures (at 35 °C and above) with high shape fidelity, retention, and resolution from an initial nonprintable ink. They stated that disruption of the hydrogen bonds of kCA polymer chains, due to the incorporation of clay nanoparticles and further bonding with the polymeric network, is the reason for the bioink’s reduced gelation points.

The viscoelastic and flow behavior properties of materials designed for 3D printing are challenging problems to address in order to obtain high-quality constructs. For this reason, Da’vila et al. [64] rheologically studied laponite/alginate mixtures to obtain an ideal ink for 3D extrusion-based printing. They observed that good-quality filament formation and printability were only achieved for laponite concentrations of at least 5 wt.%. For laponite contents lower than 3 wt.%, the printed structures collapsed and led to an inefficient printing process. The shape fidelity drastically improved with higher laponite concentrations.

### 2.4. Structural Integrity

A hydrogel scaffold for use in tissue engineering must be structurally stable in order to endure in vivo mechanical stresses while supporting the ingrowing tissue [65]. Enhanced mechanical toughness, which is due to the incorporation of nanoparticles between polymer chains causing the increase in entanglements of the polymer network, is a vital property for biomaterials used in regenerative medicine [43].

**Figure 12:**
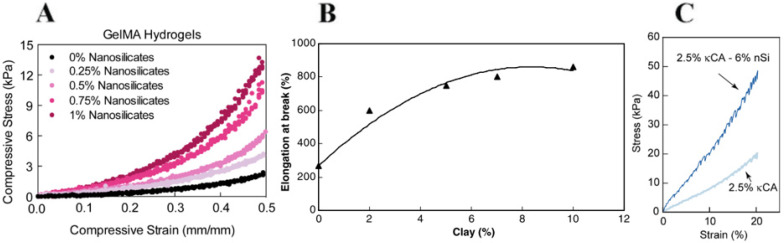
(**A**) Uniaxial compression test of GelMA hydrogels with nanosilicates’ addition. (Adapted with permission from [66]. Copyright 2018, Elsevier). (**B**) Elongation at break of polyvinyl alcohol (PVA) and organoclay nanocomposite hydrogel films. (Adapted with permission from [52]. Copyright 2017, Elsevier). (**C**) Uniaxial compression test of cross-linked 3D structures printed from κCA−nanosilicate bioinks. (Adapted with permission from [63]. Copyright 2017, American Chemical Society).

**Figure 13:**
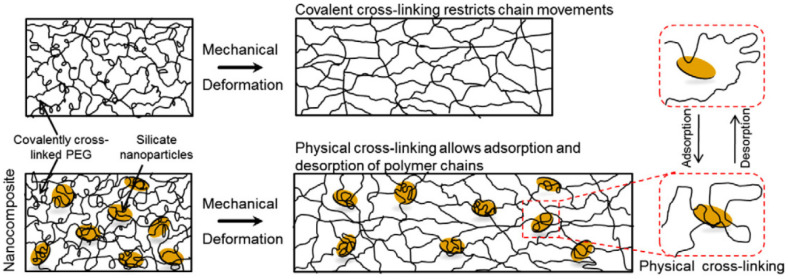
Effect of clay incorporation on the mechanical properties of the PEG network. Mechanically strong and highly elastomeric hydrogels resulted from covalent cross-linked polymeric chains and physical cross-linking between silicate nanoparticles and polymeric chains. (Reprinted with permission from [65]. Copyright 2015, Elsevier).

To mimic the native tissue interface, Cross et al. [66] produced a gradient scaffold composed of GelMA and methacrylated kCA reinforced with clay NPs, which significantly increased the strength of the hydrogels (Figure 12A). With 1 wt.% clay addition, the compressive strength of GeIMA hydrogel increased up to seven-fold and three-fold for MkCA hydrogel. Typically, when clays are incorporated into polymeric hydrogels, a diffusion of the polymeric chains to the basal space of the silicate layers occurs, creating strong interfacial interactions. Therefore, it is estimated that nanocomposite hydrogels may bear more external load than pure ones, as mentioned below. For example, Kokabi et al. [52] tested the elongation at break of PVA and organoclay hydrogel nanocomposite films as well as pure PVA films. As shown in Figure 12B, the elongation at break increases drastically with organoclay increase. By adding 10 wt.% of organically modified Montmorillonite (OMMT) to PVA hydrogel, the elongation at break of the nanocomposites increases from 270% to 860% (3.2 times), which is undoubtedly due to the presence of organoclay and its more entangled structure in comparison with pure gels. Also, the tensile modulus increased 27% with 10% clay addition. Most of the available bionks are unable to withstand physiologically related stress dynamics due to insufficient mechanical strength, among other manufacturing difficulties. For these reasons, several bioinks have been modified with clay minerals. For instance, Wilson et al. [63] studied the mechanical properties of 3D-printed structures and nanosilicate addition into kCA hydrogels. The κCA−nSi bioink formulations had superior modulus when compared to other bioinks available for use. As observed in Figure 12C, the compressive modulus for pure κCA gels was 85 ± 5.7 kPa. The addition of 6 wt.% clay resulted in an almost 2.5-fold increase to 208 ± 6.5 kPa. The mechanical properties of these nanocomposites are similar to those of some tissues exposed to mechanical stress, such as cartilage and blood vessels. In another study, Gaharwar et al. [65] evaluated the mechanical properties of PEG/laponite nanocomposite hydrogels. The results indicate the formation of physical bonds between the silicate and PEG chains, producing a more viscoelastic nanocomposite (Figure 13). Polymer chains reversibly interact with the surface of laponite particles and can adopt different conformations. They also found in the same way as above mentioned, that during mechanical deformation, these physically adsorbed polymer chains contribute to enhanced elongation. The incorporation of a silicate clay increased fracture strength, ultimate strain, elongation, toughness, and adhesion properties. An increase in the Young’s modulus was also expected with the increase of laponite concentration. They hypothesized that the disruption of PEG covalent links by the formation of reversible physical cross-links between laponite particles and PEG chains counterbalanced the variations in the Young’s modulus leading to no significant differences. 

### 2.5. Degradation

The degradability of biomaterial systems is a fundamental characteristic with significant effects on biomedical applications such as tissue engineering applications, where scaffolds should provide and maintain the needed structural support as long as the regeneration of new tissue demands. It is also an important factor for drug delivery applications in order to prevent therapeutic agents from premature burst release [67].

For bone regeneration, Paul et al. [68] introduced an extracellular matrix-mimicking nanocomposite hydrogel (GelMA reinforced with laponite). The dry weight and degradation profiles of photo crosslinked 7% GelMA hydrogels reinforced with various clay contents were assessed after exposure to collagenase enzyme for 24 h. After 24 h, the groups with 0.05% and 0.5% (*w*/*v*) of clay NPs exhibited significantly reduced degradation rates when compared to the other two groups. The same conclusions were drawn by Mauro et al. [69] which developed polyamidoamine–montmorillonite hydrogel composites for bone tissue applications and observed increased degradation times with the addition of clay nanoparticles. Gaharwar et al. [57] also observed a strong correlation between the concentration of laponite silicates and the dissolution rate of PEO-based nanocomposite films. They concluded that the cross-linking of PEO chains by silicate nanoparticles retards the dissolution rate of the films, consequently prolonging the release of entrapped proteins. In the same line of work, Kerativitayanan et al. [67] developed poly(glycerol sebacate) (PGS) nanocomposites for musculoskeletal tissue engineering reinforced with laponite nanoparticles, which also experienced significantly reduced degradation. Significant surface erosion and agglomeration of the degraded product were noticed for pristine PGS, and PGS reinforced with 1% of clay. However, the surface morphology of PGS reinforced with 10% clay nanoparticles remained pristine. They concluded that the addition of clay minerals increased the crosslinking degree, improved the degradability by retarding the weight loss rate, and enhanced the physiological stability. These studies suggest a clear correlation between the presence of clay nanoparticles and the resultant degradation improvements.

### 2.6. Morphology

Besides tuning the mechanical characteristics, the microstructure of the hydrogel’s matrix is also an important factor for mass transport mechanisms, aiding the delivery of biological agents as well as allowing dynamic interaction/exchanges with native cellular environments for the regeneration of tissues within the scaffold. Mass transport can be conditioned by pore size, shape, and free volume, and directly depends on scaffold architecture [70].

**Figure 14:**
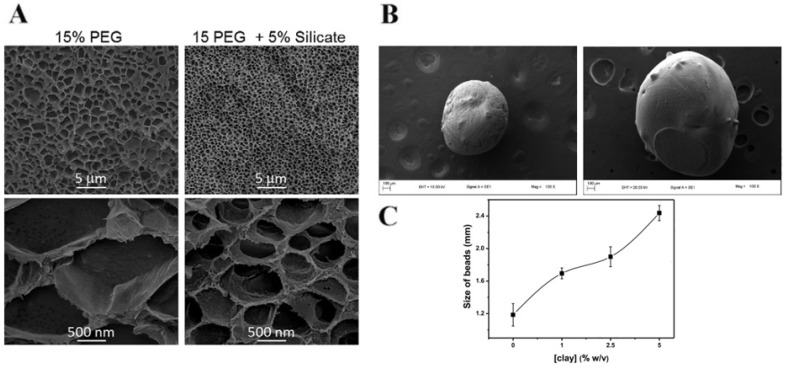
(**A**) Representative SEM images of hydrogel microstructures showing highly porous structures with interconnected pores. The addition of silicate nanoparticles decreases the pore size of hydrogel networks. (Adapted with permission from [65]. Copyright 2015, Elsevier). (**B**) SEM image of the pristine alginate hydrogel bead (left) and nanocomposite bead prepared with 1% clay (right). (**C**) Relation between the size of alginate hydrogel nanocomposite beads and clay concentration. (Adapted with permission from [50]. Copyright 2018, Elsevier).

On this point, Kokabi et al. [52] evaluated the interlaminar spaces of clay and organophilic clay as well as the structure and morphology associated with the corresponding spacing between the silicate layers in the nanocomposites produced. The increase in the interlaminar space of the samples, with the respective diffraction peak profile decrease, is due to the clay’s organic modification process and the intercalation of PVA polymeric networks within the interlayer silicates’ space during the freezing–thawing process. In a more recent study, Pereira et al. [71] studied the influence of bentonite and organically modified bentonite on the properties of hydrolyzed polyacrylamide (HPAM) composite hydrogels. The results indicated an improved interaction between the modified bentonite (organophilic clay) and the polymeric matrix, displaying a continuous network with a denser structure devoid of porosity when compared to the fragile pristine HPAM hydrogels and the porous structure of the bentonite-reinforced hydrogels (due to the low affinity to the unmodified bentonite clay). A decreased pore size due to clay addition was also observed by Gaharwar et al. [65] which developed hydrogels with a highly interconnected and porous structure ((Figure 14A). These hydrogels are composed of PEG 15% with a pore size within the range of 1–2 µm, dependent on the hydrogel composition and cross-linking density. The addition of 5% laponite silicate decreases the pore sizes to 1–0.5 µm. In an interesting and insightful work, Fernandes et al. [50] evaluated the clay concentration effect on the morphology of alginate hydrogel beads. They observed that the incorporation of nanoclays increases the surface and subsurface roughness of the beads. Additionally, this incorporation also increased the diameter of the hydrogel beads (Figure 14B,C). This effect was explained by the hydration and swelling of the nanoclay, which could generate higher expansion levels. Therefore, the roughness obtained may increase the contact surface area of the nanocomposite beads, thus increasing the absorption of water and solutes.

Other possible benefits of clay addition on nanocomposite hydrogel’s morphology and consequently future applications were discussed by Huang et al. [60]. The authors observed that the incorporation of HNTs in sodium alginate hydrogels does not change the crystalline structure of the clay, as demonstrated by the diffraction spectrum where no new diffraction peaks appear, and their location does not change. These nanocomposite hydrogels exhibited a pore size in the range of 100–250 μm, and it slightly decreased when compared with pure SA hydrogel. They stated that the hydrogel structure and pore size of 200 μm is beneficial to homogeneous cell seeding and penetration, suited for cell growth, proliferation, cell–materials interactions, and sufficient space for ECM secretion. The roughness of the pore surface was shown to be tailored by the concentration of HNTs, providing a good interface for cell adhesion. The addition of HNTs into the SA solution leads to a decrease in water content which results in the decrease in pore size during the freeze-drying process.

### 2.7. Encapsulation/Release

To design a drug release system, several parameters and properties should be analyzed; these include the processing conditions, the surface properties, the morphology, and porosity of the system. These properties can influence not only the amount of encapsulated drug, but also its release mechanism. Considering the release mechanisms from nanocomposite systems of hydrogels with clays, these can be dictated by the polymer swelling or degradation, the formation of barriers to simple diffusion, or the interactions between polymer, clay, and water [72].

**Figure 15:**
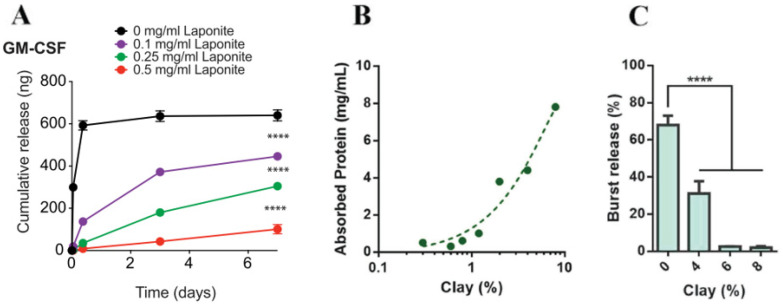
(**A**) GM-CSF release from laponite-reinforced click alginate cryogels. (Adapted with permission from [72]. Copyright 2018, Elsevier). (**B**) Clay’s influence on the loading capacity of a 8 mg/mL solution of IGF1 mimetic protein and (**C**) quantification of a 24 h measured burst release from the release profiles of composite hydrogels. Adapted with permission from [74]. Copyright 2018, John Wiley and Sons).

To analyze the effect of clay nanoparticles on the encapsulation and delivery of therapeutic agents, Hua et al. [73] prepared a series of ofloxacin/MMT/chitosan (OFL/MMT/CS) hydrogels for drug delivery applications and observed that the presence of MMT improved the encapsulation efficiency of OFL compared to beads without MMT. This might be due to the large specific area of MMT clays which adsorbs OFL both at the surface and in the interlayer space. Drug release occurred through a combination of swelling and degradation of the beads in simulated gastric fluid (SGF). The addition of MMT also provided a slower, continuous diffusion of OFL. In another approach, Koshy et al. [72] tested the ability of charged laponite nanoplatelets to pre-adsorb and impact different proteins (of varying size, net charge, and immune function) released from injectable click alginate cryogels. Injection of newly formed laponite-containing cryogels eliminated the burst release of all tested proteins and maintained their controlled release over the course of four weeks (Figure 15A). Additionally, data suggested that protein bioactivity is maintained after release from cryogels. Since clay minerals preserved their high surface area and charge density after nanocomposite assembly, Li et al. [74] were able to bind large amounts of the PEGylated IGF1 mimetic protein: 8.0 mg/mL for 8% clay as seen in Figure 15B when compared with conventional hydrogel drug loadings (range from 0.01 to 1.0 mg/mL). This was attributed to electrostatic attractions between IGF1 net positive charge and negative surface of clays. For systems with 6% and 8% clay loadings, a nearly zero-order release profile with little initial burst was observed (Figure 15C). Contrarily, alginate hydrogels without clay exhibited a significant initial burst release (> 60%), along with a less than 3-day release time. They hypothesized that since the size of the individual protein molecules is usually smaller than the network meshes of the alginate hydrogel, the presence of clay nanoparticles was significant for active control over the protein release rate. The same conclusions were observed by Gaharwar et al. [57] who determined the effect of silicates on 0.05 wt.% albumin release kinetics from laponite clay cross-linked PEO nanocomposite films. Since PEO is a water-soluble polymer, pure PEO films dissolve in PBS immediately. Therefore, the incorporation of high amounts of silicate cross-linkers creates a denser network, leading to a gradually smaller initial burst release, with the release becoming more sustained and prolonged as the silicate concentration increases. This was justified by the presence of silicate nanoparticles, which can act as obstacles to macromolecular protein diffusion through the creation of a denser network with narrow paths.

### 2.8. Cell Dynamics

Interaction and adhesion of cells (such as mesenchymal stem cells) to biomaterials is one of the important parameters to control, since it is vital and relates to various cell behaviors such as cell survival, proliferation, and differentiation. For instance, the absence of cell adhesion sites in PEG hydrogels results in considerably reduced cell viability. Incorporation of low amounts of clay nanoparticles exhibited round and spherical hMSCs morphology due to poor interaction between the cell and the polymeric matrix while, at higher amounts, a well-organized cytoskeleton is presented. Thus, a strong connection between the concentration of clay nanoparticles and cell adhesion, spreading, and cytoskeletal organization was noted. For the nanocomposite PEG chains, their hydration results in the exposure of silicate surfaces, known to act as cell adhesion sites [75]. Also, incorporation of laponite to PEG films to control cell adhesion is a simple, highly cost effective and reproducible procedure.

**Figure 16:**
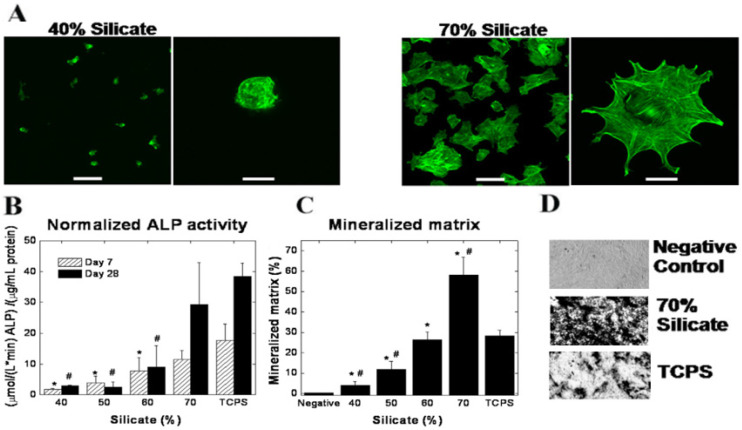
(**A**) Representative confocal imagens of preosteoblast cells at the PEO nanocomposite surface. F-actin was stained to demonstrate the enhanced cell adhesion, spreading, and proliferation caused by the presence of laponite. (**B**) Quantification of ALP activity. A 6-fold increase in ALP activity was observed on day 7 and a 10-fold increase on day 28 when silicate concentration increases from 40% to 70%. (**C**,**D**) Qualitative and quantitative evaluation of mineralized matrix after 28 days with positive control (TCPS). (Adapted with permission from [57]. Copyright 2011, Elsevier).

For cell adhesion, Gaharawr et al. [57] developed and studied cross-linked PEO films containing different amounts of laponite to induce the spreading and proliferation of MC3T3-E1 mouse preosteoblast cells and matrix mineralization. The authors observed that, using pure PEO films, no protein adsorption was observed, thus compromising cell adhesion and proliferation. However, the incorporation of higher amounts of laponite improved cell adhesion and spreading, thus leading to significant changes in the cytoskeleton organization. With lower concentrations of laponite, the F-actin fibers were not formed, appearing disrupted and disorganized. Consequently, cells exhibited a spherical morphology which indicates the lack of adhesion points between the nanocomposite surface and cells. With high concentrations of laponite, the F-actin fibers were clearly visible, with cells displaying a well-spread flat morphology and thick bundles that were stretched inside the cells. Therefore, cells were able to develop protruding cellular arms, thus forming connections between adjacent cells, enhancing cell adhesion and proliferation at the nanocomposite surface (Figure 16A). They observed that by increasing the concentration of laponite from 40% to 70%, cell adhesion and spreading increased 4-fold and 3-fold, respectively. Additionally, cell viability was not affected by the concentration of laponite incorporated in the nanocomposite. The authors also studied the activity of the osteoblast cells using alkaline phosphatase (ALP) as an early-stage marker of osteoblast phenotype and calcified matrix production (Figure 16B). This parameter significantly increased with the concentration of laponite, demonstrating the relationship between cell adhesion (and morphology) and osteoblast activity. These results were confirmed by von Kossa staining to visualize the production of phosphate by osteoblast cells in order to compose the ECM. They observed the same silicate concentration-dependent behavior, and a 30-fold increase in the amount of mineralized ECM was observed when the concentration of laponite increased from 40% to 70% (Figure 16C,D).

In another study, Dong et al. [76] developed a laponite-reinforced gelatin methacrylate cross-linked hydrogel ink for 3D printing and bone regeneration. Cells were able to adhere and proliferate at the composite hydrogels surface. Additionally, the osteogenic differentiation of BMSCs in vitro was also observed. This support function was achieved due to improved protein adsorption by electrostatic interactions with laponite, thus improving cell adhesion and proliferation. The addition of nanoclay significantly enhanced the ALP activity. To evaluate the late osteogenic differentiation, Alizarin Red S (ARS) was used to detect/evaluate the deposition of calcium nodules in cell cultures. These results also confirmed a direct relationship between the laponite concentration and cell differentiation and calcium deposition. A higher expression of osteogenic genes when 8% of laponite is incorporated in GelMA hydrogels was observed. These results demonstrate that, by adding a high concentration of laponite, it is possible to induce cell differentiation as well as being possible to develop a bone regeneration scaffold with reduced costs and without delivering growth factors. Laponite demonstrated osteoinductive properties due to its dissociation, which releases magnesium ions (Mg^2+^), lithium ions (Li^+^), and orthosilicic acid (Si(OH)_4_). This release increases the adhesion and stability of cells which leads to ossification and increased ECM production.

## 3. Fields of Application

### 3.1. Tissue Engineering

The ability to replicate native environment dynamics and functions is the main objective of tissue engineering (TE) applications for the development of well-designed functional biological substitutes (Table 1). There is a wide range of cell-incorporated 3D scaffolds that can be delivered to damaged sites by physiological integration or used as platforms and structural support, while enabling the exchange of nutrients and oxygen for the regeneration and formation of new tissue [77].

The requirements for the development of material scaffolds for TE applications include controllable physical and chemical properties, predictable biological responses, and the capacity to adapt to the biological environmental conditions. However, these conditions are quite challenging due to the variety and complexity of biological tissues. These can include soft tissue regeneration, where flexible and soft nanoengineered materials are required, and hard tissue regeneration, where tough load-bearing materials are needed. For optimal functionality there are several requirements that all scaffolds should be able to satisfy, such as (i) biocompatibility of the manufacturing process and what it entails; (ii) scaffolds’ ability to endure mechanical stress, maintain structural integrity and functionality as long as required; (iii) morphological and structural characteristics that ought to promote cellular adhesion, enable integration and interaction with the biological microenvironment; and (iv) highly porous microstructure to allow cellular migration and proliferation, ingrowth of surrounding tissue, and angiogenesis [3].

### 3.2. Bone Tissue Engineering

There are several possible causes for the occurrence of bone defects, such as physiological deformities and forced trauma, among others. An important role in the rehabilitation of patients and reconstruction of bone defect is the appropriate selection of suitable and well-designed scaffolds, with 3D constructs that fit the original surroundings and emulate the local environment. Different degradable and nondegradable scaffold composites have been studied for bone tissue engineering (Table 2) [54].

**Figure 17:**
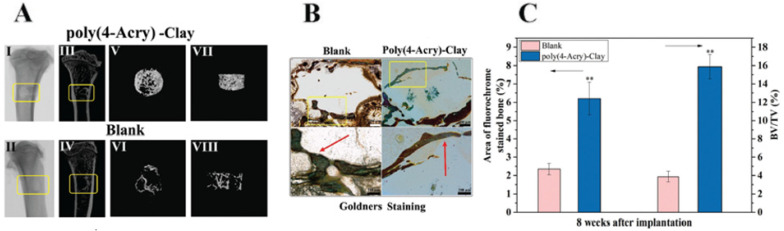
(**A**) Micro-CT characterization of implant and new bone formation (I and II), reconstruction images (III and IV) and reconstructed 3D models (V to VIII). (**B**) Histological staining observations of new bone formation, shown by the red arrows, 8 weeks after surgery for poly(4-acry)–clay nanocomposite hydrogel with a 5% nanoclay concentration. (**C**) Relative bone formation assessment 8 weeks after implantation. (Adapted with permission from [81]. Copyright 2013, Royal Society of Chemistry).

For bone defect regeneration, Zhai et al. [81] developed a poly(4-acryloylmorpholine) laponite-based nanocomposite hydrogel with bioactive ion release. It exhibited a suitable environment for cell growth and excellent mechanical properties. Due to the gradual release of the clay’s intrinsic Mg^2+^ and Si^4+^ ions, the nanocomposite hydrogel supported ALP production by primary rat osteoblasts (ROBs). Therefore, following implantation in vivo, new bone formation is stimulated. Micro-CT analysis and reconstructed 3D images demonstrated that nanocomposite hydrogel with 5% of clay stimulated bone growth in the tibia defect in comparison with control (Figure 17A). These results were also demonstrated by Goldner’s stained histological sections (8 weeks after surgery), where newly formed bones are much thicker and continuous around the implanted hydrogel and have been fully mineralized (stained in green), further verifying that the nanocomposite hydrogels can facilitate new bone formation (Figure 17B,C). Additionally, the absence of macrophages or foreign body reaction giant cells in histologic analysis demonstrated no apparent inflammation or other adverse reaction.

The development of 3D cell-culture scaffolds that can mimic native tissue conditions has also been improved by nanoclay addition, which confers the ability to produce superior microfabricated structures. This requires the production of cell-friendly biomaterials, high drug loading, and fast and efficient self-healing ability, among other things. Biomaterials used as bioinks for 3D printing and bone regeneration should fulfill these principles: (i) suitable rheological behavior and extrusion processability for 3D-bioprinting, (ii) the printed construct must be stable without collapsing before solidification or crosslinking, (iii) high cell viability and suitable structure porosity for the diffusion of nutrients and oxygen, (iv) proper and suitable mechanical characteristics, and (v) osteogenic differentiation and long term new bone formation [82]. 

**Table 2:** Clay nanoparticles in hydrogel nanocomposites for bone tissue regeneration.

Hydrogel	Clay	Features/Observation	Ref
Chitosan glycerophosphate	HNT	Icariin (IC), a bone inducer, was loaded into modified-HNTs (mHNTs), resulting in a sustained drug release system; Modification of HNTs with chitosan increased entrapment efficiency and loading capacity with reduced initial burst release of IC. The nanocomposite chitosan/mHNTs displayed decreased gelation time and temperature, enhanced mechanical strength, improved proliferation, and bone differentiation of hASCs and hMSCs encapsulated in the resulting scaffolds.	[83]
Poly(4-acryloylmorpholine)	Laponite	The clay-based nanocomposite displayed excellent mechanical properties, good biocompatibility, sustainable release of bioactive ions (intrinsic Mg^2+^ and Si^4+^), ability to promote osteogenic differentiation of ROBs and effective formation of new bone after implantation.	[81]
PNAGA	Laponite	Implantation of 3D-printed scaffold showed sustainable release of intrinsic ions and osteogenic differentiation of ROBs, which facilitated the regeneration of new bone in tibia defects of rats.	[54]
GeIMA	nSi	Nanoengineered collagen-based matrix reinforced with nanosilicates displayed osteogenic differentiation (in absence of any osteoinduction factors), and improved migration and proliferation of hMSCs.	[68]
AGMA1	MMT	Fully swollen AGMA1–MMT hydrogels showed storage modulus (G′) values up to 20 times higher than common biomimetic hydrogels; The nanocomposite was completely degradable with no cytotoxicity, supported cell adhesion and proliferation and induced osteogenic differentiation of mouse calvaria-derived pre-osteoblastic cells (MC3T3-E1).	[69]
Collagen-based GelMA	Laponite	Clay nanoparticles enhanced the formation of mineralized matrix (in a growth factor-free environment); Laponite addition increased porosity, improved scaffolds stiffness (four-fold increase in compressive modulus) and injectability.	[84]
Silk fibroin	Laponite	The composite hydrogel can be recovered within 70 s after shearing with 3000% strain, for at least 6 successive repeats. Incorporation of laponite nanoparticles promoted osteogenic differentiation of primary osteoblast in the regenerated silk fibroin-based scaffold.	[62]
PEGDA	Laponite	The lamellar and porous nanocomposite hydrogel exhibited in vivo bone healing capacity with the formation of intramembranous bone in a defect model of the tibiae of osteopenic rats. The *Artemia salina* lethality assays demonstrated no toxicity.	[85]

HNT—halloysite nanotubes; PNAGA—poly (N-acryloyl glycinamide; GelMA—Gelatin methacryloyl; nSi—disk-shaped nanosilicates; AGMA1—peptidomimetic polyamidoamine carrying guanidine pendants; MMT—montmorillonite; PEGDA—polyethylene-glycol diacrylate; hASCs—human adipose-derived stem cells; hMSCs—mesenchymal stem cells.

**Figure 18:**
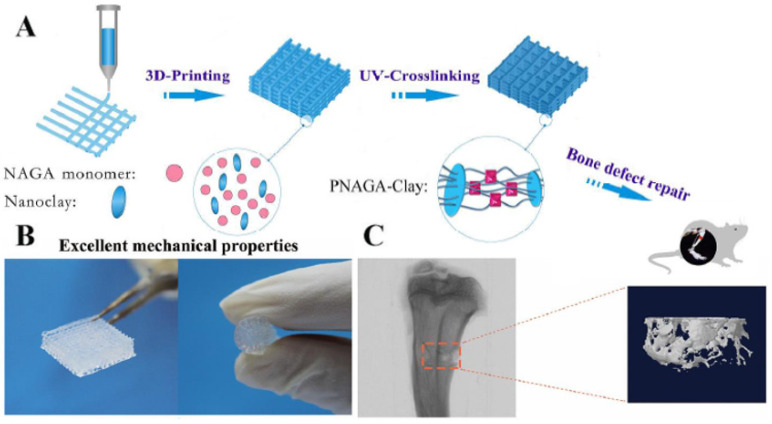
(**A**) 3D-printed PNAGA–clay scaffold procedure. (**B**) Scaffold’s capacity to resist finger compression. (**C**) Micro-CT characterization of implant and new bone formation and reconstructed 3D model of the new bone. (Adapted with permission from [54]. Copyright 2017, American Chemical Society).

In this regard, Zhai et al. [54] developed a nanoclay-based bioink using a hydrogen-bonding monomer (N-acryloyl glycinamide) (NAGA). The 3D load-bearing polymer/clay nanocomposite hydrogel scaffold was printed with an adequate architecture to precisely repair individual bone defects (Figure 18A). The high strength (complete recovery of the scaffold’s microstructure following car wheel compression test) and swelling stability of the printed construct (maintained stable original architecture for 3 months in water without any delamination of the 3D scaffold’s 10 layers), ensures its load-bearing capability as well as its reliable microstructural integrity (Figure 18B), which is crucial for in vivo bone regeneration. Similarly to other clay-based scaffolds, PNAGA-clay nanocomposite sustainably released Mg^2+^ and Si^4+^ ions, which influenced cell behavior and promoted a higher gene expression of ALP, OCN, and collagen after 7-day culture. The penetration of the new bone formed inside the microstructural holes of the scaffold confirms the excellent osteoconductivity of PNAGA–clay nanocomposites. The stimulated new bone volume around and inside the nanocomposite scaffold in the marrow cavity (5.5%) was shown by the reconstructed 3D models (Micro-CT images seen in Figure 18C) to be larger and much thicker that the blank control group (2.8% without intervention).

Other materials developed to aid bone regeneration include physically cross-linked and injectable double-stranded DNA hydrogels reinforced with laponite. These hydrogels showed controlled release of dexamethasone, an osteogenic drug, thus leading to new bone formation in in vivo results using rats with cranial defects [86]. Another study developed a combinatorial approach of alginate, hyaluronic acid, and 8-arm PEG acrylate reinforced with laponite and montmorillonite. This composite material demonstrated a 36-fold increase in ALP activity and an 11-fold increase in mineralized matrix growth compared to control [87]. An injectable laponite-reinforced alginate hydrogel demonstrated improved compressive strength, water uptake, and viscosity. Additionally, osteoblasts’ viability was enhanced compared to alginate alone [88]. Another example includes a hydrogel reinforced with silicate nanoparticles as a coating for titanium implants. This nanocomposite hydrogel showed adhesive, osteoconductive, and antimicrobial properties with the potential to promote new bone formation in the surrounding tissues [89]. A more recent study developed 3D-printed gelatin methacryloyl (GelMA) hydrogels reinforced with laponite in order to increase mineralization capability for bone repair and efficiently bind and deliver bioactive factors and tools such as epigenetically enhanced extracellular vesicles to stimulate bone regeneration [90].

### 3.3. Cartilage Tissue Regeneration

Cartilage is a flexible connective tissue of specialized cells called chondrocytes (which secrete collagenous ECM to maintain and sustain the cartilage) that are dispersed into highly organized collagenous extracellular matrices abundant in proteoglycans, glycosaminoglycans (GAGs), and collagen fibers [70]. The repair of damaged cartilage tissue remains an orthopedic challenge due to the reduced mobility of chondrocyte in the ECM as well as the absence of progenitor cells and decreased blood and nutrient supply (vascular networks). Among the numerous multiphasic implants tested in recent decades for cartilage regeneration, clay-reinforced hydrogels have been widely researched (Table 3), due to their ability to mimic the physicochemical and biological properties of native hydrated cartilage ECM and promote integration and scaffold–cell interactions [70].

There have been in situ studies of the formation of injectable hydrogels and scaffold constructs for cartilage repair and structural support in vulnerable load-bearing cartilage tissue. Some examples are thermo-responsive injectable hydrogels composed of wool-derived fibrous protein (keratin), triblock copolymer (pluronic), and chitosan reinforced with laponite clay, exhibiting improved elastic modulus and biological stability, without compromising the in vitro cell viability, as well as enhanced cell adhesion and proliferation [70]; and LDH clay added to polypeptide thermo-responsive gels for chondrogenic differentiation of stem cells on damaged cartilages. These nanocomposite gels improved cell aggregation with enhanced the expression of chondrogenic biomarkers (mRNA and protein levels) compared to control [91].

**Table 3:** Clay nanoparticles in hydrogel nanocomposites for cartilage repair.

Hydrogel	Clay	Features/Observation	Ref
GG + antibacterial MH	HNT + BE + MS	Incorporation of inorganic clays significantly improved the mechanical properties. No severe immune response was caused, and the infection was restrained; MS provided the best combination in terms of in vitro cytocompatibility, mechanical performances, and morphological features; GG-MH-MS tested in co-culture preserved hMSCs’ proliferation over bacteria.	[92]
MκCA	2D nanosilicates	Nanocomposite hydrogel as an injectable for cellular delivery; The strong interaction between polymer chains and nanosilicates resulted in nanocomposites with shear-thinning characteristics, reinforced hydrogel networks, elastomeric properties, physiological stability, and high cell viability after injection.	[93]
PEG	Laponite	The presence of silicate enhanced bioactivity, cell adhesion, spreading, and growth as well as the adhesiveness of the hydrogel to soft tissue and hard surfaces.	[65]
Si-HPMC	Laponite	Interpenetrating network enhances the hydrogel mechanical properties without interfering with its cytocompatibility, oxygen diffusion, or the ability of chondrogenic cells to self-organize in the cluster and produce ECM.	[94]
pNIPAAM	LDHs	pNIPAAM-based hydrogel capable of delivering siRNA using LDH platelets. The injectable and thermo-responsive properties of the hydrogel allowed it to be administered locally in a minimally invasive manner, thus providing a strategy for the in vivo treatment of degeneration in cartilaginous tissues.	[95]
PEO	Laponite	An increase in mineralized phosphate produced on the bioactive nanocomposite surfaces indicated that the silicate nanoparticles influence the differentiation of preosteoblast cells.	[57]
PEG	Laponite	The nanocomposites exhibited biocompatibility, scaffold ability to withstand load and stress from native cartilage (collagen type II), due to clay nanoparticle exfoliation, and enhanced storage modulus.	[96]

GG—gellan gum, MH—manuka honey; HNTs—halloysite nanotubes; BE—bentonite, MS—mesoporous silica; MκCA—methacrylated kappa-Carrageenan; PEG—Poly(ethylene glycol); Si-HPMC—silated hydroxypropylmethyl cellulose; pNIPAAM—poly(N-isopropylacrylamide; LDHs—layered double hydroxides; PEO—poly(ethylene oxide); hMSCs—human mesenchymal stem cells.

### 3.4. Drug Delivery

Drug delivery systems mainly aim to solve one of the biggest issues in the conventional treatments of many diseases: the poor delivery of drugs to the target sites causing undesired side effects. It is desired to maintain drug levels within the therapeutic concentration range during the course of treatment. This is possible by using delivery systems that are able to release a drug at the target site in a controllable manner (Table 4). Additionally, the incorporation of the drug in the delivery system protects the drug from rapid degradation while enhancing its action in the target tissue for longer periods with adequate release kinetics, which requires lower and less-frequent doses [97]. 

**Figure 19:**
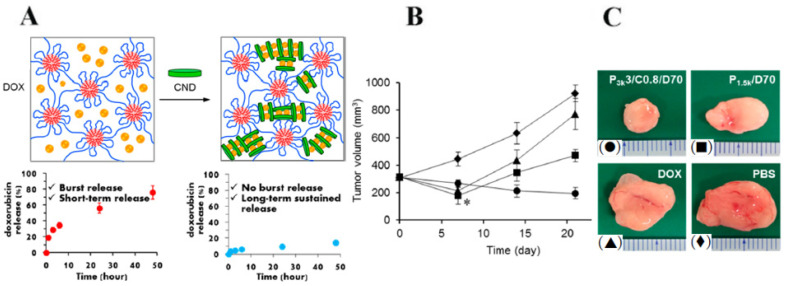
(**A**) Representation of PLGA-PEG-PLGA/DOX copolymer gel and PLGA-PEG-PLGA/CND/DOX hybrid gel. (**B**) In vivo antitumor activity of DOX-loaded hybrid gels against human cancer cells implanted in mice. PBS as control (◆); DOX solution (▲); P_1.5k_/D70 gel (■); and P_3k_3/C0.8/D70 gel (0.8 wt.% clay) (●) and (**C**) photographs of the excised tumors from animals sacrificed 21 days after injection. (Adapted with permission from [98]. Copyright 2015, American Chemical Society).

The use of biodegradable injectable gels for localized drug delivery is a promising treatment method. Nagahama et al. [98] proposed the fabrication of a safe and effective biodegradable injectable gel based on self-assembling PLGA-PEG-PLGA copolymer micelles and clay nanodisks (CNDs) for doxorubicin (DOX) delivery and local treatment of cancer. From a single injection of hybrid gel, they achieved no initial burst release, as well as long-term sustained release of DOX, and provided sustained antitumor activity in vivo (against human xenograft tumors in mice) (Figure 19A). The tumor volume (300 mm^3^) increased almost 3-and 2-fold after 21 days while treated with just PBS and free DOX. In contrast, a gradual decrease in tumor volume was observed in mice treated with P3k/CND/DOX hybrid gel (Figure 19B,C) without any associated dermatitis.

For tendon repair, Li et al. [74] proposed an extended local release of an insulin-like growth factor-1 (IGF1) mimetic protein, conjugated with PEG, laponite and a biodegradable alginate hydrogel network. The hydrogel scaffolds were surgically implanted into the Achilles tendon injury and released IGF1 over long periods of time, exposing the damaged tendon to a concentration four orders of magnitude higher compared to simple injection, with localized and minimal entry of the drug into the circulation. Analysis of histological sections (distribution of the fluorescein-labeled protein) after 8 days of scaffold implantation, revealed localized protein distribution in the tendon and degradation into multiple smaller fragments within one week.

Many other studies have been published on the influence of different types of clay and their effects on the drug-release profiles of diverse hydrogels for different therapies: hydrotalcite clay intercalated with different kind of drugs [99]; influence of laponite exfoliation on the release profile of thermo-sensitive hydrogels [100]; magnetic-dependent drug release due to immobilized Fe_3_O_4_ nanoparticles on laponite clays [101]; LDH clay and montmorillonite influence on vitamin B12 release [102] and in artificial gastric fluid (AGF) and artificial intestinal fluid (AIF) [103]; laponite’s influence on the sustained controlled release of DOX for cancer therapy in a physiological environment [104]; laponite clay–polymer microbeads for anti-inflammatory effect in rats by oral controlled drug delivery of diclofenac sodium [105]; the encapsulation and release behavior of inorganic organoclay MMT-based hydrogels for three different model drugs, and the clay’s influence on cytotoxicity and biodegradation rates in different conditions [106]; antimicrobial and pH-responsive nanocomposite hydrogels based on sepiolite clays for the delivery of ceftriaxone sodium antibiotic used in the treatment of infections [107] and preparation of gelatin-based aerogels reinforced with MMT for diltiazem delivery, a drug used to reduce blood pressure [108].

**Table 4:** Clay nanoparticles in hydrogel nanocomposites for drug delivery applications.

Hydrogel	Clay	Features/Observation	Ref
Chitosan	MMT	MMT addition to pristine chitosan hydrogel beads enhanced their stability and disintegration performance, improved the swelling behavior, increased ofloxacin entrapment, and promoted a sustainable drug release profile.	[73]
(PLGA-PEG-PLGA)	CNDs	A single injection of the biodegradable gel provided sustained DOX antitumor activity in nude mice and significant tumor reduction compared to control.	[98]
Alginate	Laponite	The addition of laponite nanoparticles substantially hindered the burst release of the adsorbed protein cargo from alginate cryogels and exhibited sustained release kinetics when compared with unreinforced hydrogels.	[72]
Sodium alginate	Laponite	The nanocomposite hydrogel exhibited increased encapsulation of IGF1 mimetic protein, biocompatibility with surrounding environment, sustained and localized drug release for tendon injury with simultaneous biodegradability.	[74]
Gelatin methacrylate	Laponite	Nanoclay incorporation in hydrogel improved the ability to modulate the release of key growth factors (present in stem cell derived secretome) and provide angiogenic and cardioprotective therapeutic ability.	[109]
HEMA	MMT	Compared to pristine HEMA hydrogels, the burst release of paracetamol from the HEMA/MMT nanocomposites was significantly reduced and its release time increased.	[110]
Sodium hyaluronate + HEMA	HNT	5-FU was encapsulated successfully into these hydrogels as well as inside the halloysite nanotubes through equilibrium swelling. The nanocomposite hydrogel exhibited pH sensitivity, uniform stabilization ability of the HNT in the hydrogel networks, 5-FU release in the gastric region and in the intestinal fluid in a controlled manner for over 70 h.	[111]
CMC	MMT	Nanocomposite hydrogel beads, composed of propanol intercalated clays and CMC, exhibited sustained release and high bead stability against simulated stomach acid and intestinal conditions.	[112]
CS + AA + AAm + PVP	MMT	A sustained drug release profile was obtained in the presence of MMT nanoparticles, maintaining clarithromycin concentration in a simulated gastric environment for prolonged periods of time.	[58]
PAAm	Laponite	Polyampholyte hydrogels reinforced with laponite were characterized for their sensitivity to external conditions and their ability to electrically control the release of the active paracetamol drug agent	[113]
CMC-g-PAA	Organo-MMT nanoclay	The presence of clay nanoparticles enhanced mechanical properties and in vitro blood compatibility. The release behavior of vitamin B_12_ was dependent on the nanocomposite cross-linking density and medium pH.	[51]

PLGA-PEG-PLGA—poly(D,L lactide-co-glycolide), poly(ethylene glycol); MMT—montmorillonite; CNDs—clay nanodisks; HEMA—poly(2-hydroxyethyl methacrylate); HNTs—halloysite nanotubes; CMC—carboxymethyl cellulose; CS—chitosan, AA—acrylic acid, AAm—acrylamide; PVP—polyvinylpyrrolidone; CMC-g-PAA—carboxymethylcellulose-g-poly(acrylic acid); IGF1—insulin-like growth factor-1; 5-FU—5-fluorouracil.

### 3.5. Wound Healing

A wound can be described as a break in the epithelial integrity of the organ that may be accompanied by disruption of the structure and function of the underlying normal tissue. To recover the integrity of the first line of defense of the human body and heal the wounded tissue, a dynamic biological process takes place. The body’s initial response to injury is hemostasis and inflammation which includes promotion of platelet adhesion to control bleeding and elimination of foreign agents. To replace the damaged tissue and develop a new one, growth factors promote the migration and proliferation of, mainly, fibroblasts and endothelial and epithelial cells to the injured area. The reepithelization of the wound occurs by cell migration together with wound contraction by myofibroblasts. Throughout maturation, the final phase of healing, the reduced vasculature and growth of collagen fibers enhances the tensile strength of the reconstructed tissue [33].

**Figure 20:**
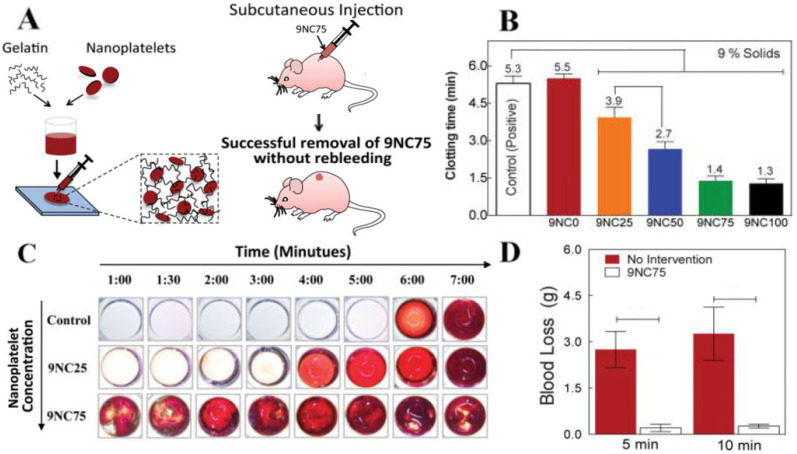
(**A**) Preparation schematic of the hemostat nanocomposite gels, rat model subcutaneous injection, and posterior removal. (**B**) Effect of nanoplatelet addition on the blood clotting time. Quantitative clot times for 9% gelatin nanocomposites. (**C**) Clot formation as a function of time and nanocomposite composition (increasing nanoplatelet content). (**D**) Effective blood loss prevention relative to the untreated hemorrhage. (Adapted with permission from [114]. Copyright 2014, American Chemical Society).

The necessity for rapid and effective regeneration of injured skin has stimulated research into advanced therapies to control the environment for wound healing (Table 5). Gaharwar et al. [114] developed shear-thinning nanocomposite hydrogels as a wound healing hemostatic agent for incompressible wounds (Figure 20A). Clay nanoparticles were incorporated in gelatin, which improved the injectability, physiological stability, nanocomposite-clot strength, and hemostatic performance by reducing blood clotting time in a dose-dependent manner (Figure 20B). For higher nanoplatelet concentrations (25%, 50%, and 75%), the clotting time decreased by 32%, 54%, and 77%, respectively, compared to control (Figure 20C). This effect can be explained by the increased adsorption of clotting factors at the surface of the nanocomposite due to the strong negative charge of clay nanoparticles. Lethal liver bleeding experiments in rats showed that the blood loss after 5 and 10 min was drastically reduced and stopped by the application of a small amount of 9NC75 nanocomposite (200 μL) (Figure 20D). Also, the remaining superficial part of the injected 9NC75 composite was shown to be easily removed without causing rebleeding.

Angiogenesis stimulation remains a major clinical challenge which can be overcome by the introduction of nanosilicate clays as a vehicle to sequester and deliver various proangiogenic growth factors. Howell et al. [115] incorporated laponite loaded with proangiogenic proteins (e.g., vascular endothelial growth factor, fibroblast growth factor, and platelet-derived growth factor) within collagen type I hydrogels to stimulate endothelial cell invasion in a 3D in vitro model of angiogenesis. They observed a robust endothelial cell sprouting/invasion/penetration into nanosilicate-loaded collagen hydrogel when compared to samples without clay, thus illustrating the capacity of clay minerals to attach proangiogenic proteins and stimulate angiogenesis.

Muco-/Bioadhesion to surfaces such as soft tissues enhances the potential of nanocomposite hydrogels as a wound dressing material due to the acquired surface roughness, attributed to the addition of clay minerals as well as the capacity to interlink with the irregularities of the surface. There have been many other clay-based nanocomposite hydrogels studied for wound healing applications [116] such as nanostructured hydrogel membranes based on chitosan biopolymer and MMT nanoparticles, with cytocompatibility, better cell morphology and attachment, and significant antibacterial activity [117]; attapulgite-reinforced hydrogel membranes where the increase in clay contents showed antimicrobial activity against six tested pathogen strains and adequate hemolytic behavior compared to clay-free membranes [118]; fibrous nanoclay and spring water hydrogels accelerated wound healing with respect to the control [119] and released therapeutic agents with beneficial activity in wound healing [120]; agar/κ-carrageenan hydrogels reinforced with MMT exhibited antibiotic and analgesic effects presenting good antibacterial activity against *E. coli* and *S. aureus* [121]; laponite-reinforced gellan gum methacrylate (GG-MA) for the treatment of burn wounds using wound-dressing material with ofloxacin release [122]; and collagen-based hydrogels reinforced with different types of clays to determine their antimicrobial behavior, cellular viability, and gentamicin delivery profiles for skin regeneration [123].

**Table 5:** Clay nanoparticles in hydrogel nanocomposites for wound healing applications.

Hydrogel	Clay	Features/Observation	Ref
PVA	Laponite	Incorporation of small amounts of laponite enhanced the rheological and mechanical properties, with a high capacity of structural regeneration (about 90%) after applying a large deformation, high water absorption capacity (900%) and improved antibacterial activity; Rifampicin release mechanism from hydrogels is dependent on clay concentration.	[124]
Gelatin	Laponite	By reinforcing gelatin biopolymers with laponite nanoplatelets, injectable hemostatic agents were developed to treat incompressible wounds in emergency circumstances; Clay particles conveyed physiological stability, rapid mechanical recovery, and ability to promote coagulation, thus being a stable clot-gel systems (decreased in vitro blood clotting time by 77%).	[114]
PVA	Na-MMT	Clay nanoparticles act as barriers against microbe penetration which enhances the protection against further wound infection and accelerates the wound healing process.	[52]
Silk sericin + pNIPAAm)	Lithium magnesium silicate hydrate	Compared with gauze, the nanoclay crosslinked nanocomposite hydrogel accelerated wound healing, hence having extensive applications in clinical medical wound dressing.	[125]
Polyacrylamide	Dopamine intercalated Clay nanosheets	The adhesive composite hydrogel exhibited resilience, strong adhesion, high stretchability, easy removal without hurting skin (allowing on-demand removal), favorable cell adhesion and proliferation as well as full-thickness skin wound regeneration.	[126]
Poly(2 methoxyethyl acrylate-co-N,N-dimethylacrylamide)	Hectorite	Due to their thermo-sensitivity, controllable modulus, and surface properties, the nanocomposite gels were promising cell culture substrates. Nanocomposite film hydrogels demonstrated excellent soft and flexible tensile strength, high elongation (more than 1000%) without suppressing the transparency.	[127]
PAM	MMT	The ozone-loaded nanocomposite hydrogels exhibited long-term antibacterial activity, protection against skin infection, and a favorable environment for wound healing.	[128]

PVA—poly(vinyl alcohol); PAM—polyacrylamide; MMT—montmorillonite.

### 3.6. 3D Bio Printing

Cell behaviors (morphology, function, fate) are deeply affected by native microenvironment characteristics, such as the physiological three-dimensional arrangement they are situated in. The aim of 3D bioprinting is to produce 3D scaffolds that can mimic the composition, architecture, and mechanical properties of native tissues as well as regenerate functional tissues and support cell–ECM interactions [129]. The incorporation of clay nanoparticles has been shown to efficiently improve the development of nanoengineered bioinks [130], enhancing their flow behavior, shape recovery, and biological activity (Table 6) [131].

**Figure 21:**
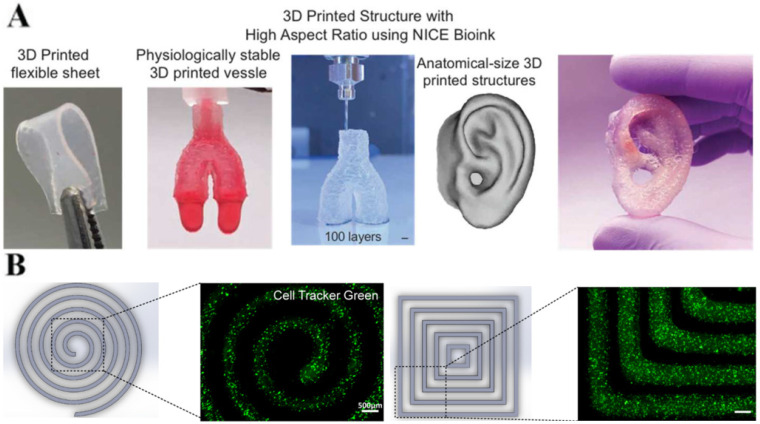
(**A**) Stable cross-linked 3D-printed structures from NICE bioinks able to support more than 50 times their own weight, have high aspect ratios and high structural fidelity (bifurcated vessels and physiological structures such as human ear). (Adapted with permission from [131]. Copyright 2018, American Chemical Society). (**B**) Bio-printed structures using κCA−nanosilicate bioinks reveal cell distribution in patterned constructs (as shown in fluorescent images using green CellTracker). (Adapted with permission from [63]. Copyright 2017, American Chemical Society).

To obtain 3D-printed anatomical structures with suitable functionality and physiologically bioactive characteristics, Chimene et al. [131] developed nanoengineered ionic covalent entanglement (NICE) bioinks comprised of bioactive GelMA and κCA ICE networks reinforced by laponite nanosilicates. The main purpose was to fabricate mechanically stiff/tough, elastomeric, and highly printable 3D biostructures (Figure 21A). The hybrid bioink formed high aspect-ratio constructs with approximately 3 cm and 150 layers tall while exhibiting excellent shape fidelity such as trachea-like bifurcated vessels and human ear. Biostructures containing murine 3T3 preosteoblasts displayed long-term viability throughout the 120-day culture observation period. After 30 days, the cell-printed biostructure turned from translucent to slightly opaque, due to deposition of ECM by the encapsulated cells and favorable nutrient transport. To develop a sustainable microenvironment for cell behaviors, Wilson et al. [63] produced a thermo-reversible hydrogel bioink composed by κCA and 2D nSi that also provided printability and yielded shape retention characteristics as well as mechanical durability. The main purpose of a bioink was to maintain the viability of incorporated cells after printing. Using green CellTracker prior to loading MC3T3-E1 mouse preosteoblasts into the bioink showed an even distribution with good viability over time in the printed 3D structures (Figure 21B).

### 3.7. Biosensors and Actuators

Stimuli-responsive hydrogels combined with nanoclays with multifunctional characteristics can also be used as biosensors and soft robotic actuators, given that their properties can be triggered by temperature, pH, and light as well as magnetic and electric fields [28]. 

In this category of stimuli-induced applications, Gao et al. [133] reported on robust poly (N-isopropylacrylamide-co-N-[3-(dimethylamino)-propyl] methacrylamide)/MMT hydrogel bilayers able to respond to both pH and temperature changes, resulting in reversible and repeatable curling/uncurling. At pH 2, it took 23.5 min to bend the bilayer into a circle and grasp a ring. Subsequently, the bilayer was transferred into a pH 12 solution, and it uncurled to release the ring in 64 min. The same shape-morphing behavior was observed when adjusting the temperature from 60 °C (grabbing) to 22 °C (releasing). In another study, Yao et al. [134] were also able to produce hydrogel strips with repeatable thermo-responsive behaviors that change their shape in water by switching the temperature rapidly between 24 and 42 °C (bending upon increasing the temperature from 24 to 42 °C and unfolding when switching the temperature back to 24 °C). Such biomimetic actuations of bilayer hydrogels may have the potential for applications as soft robotic actuators, sensors, and drug-delivery vehicles. Another system by Tan et al. [135] evaluated the potential of laponite-crosslinked (N,N-diethylacrylamide)-co-(2-dimethylamino) ethyl methacrylate hydrogels as temperature switches to change the optical transparency. As the temperature gradually increased, from 20 °C to 40 °C, the transparency of the nanocomposite hydrogels gradually decreased until slightly opaque. This was the first report of using colloidal laponite to change the visual gradient of thermo-responsive nanocomposite hydrogel. Other studies on clay-based multifunctional hydrogels for biosensor, robotic, and actuator applications and wearable devices have been published: temperature/pH sensitivity and magnetic-field-sensitive based on attapulgite clay [136]; anisotropic thermo-responsive property [137], and stretchability, conductivity with light/thermo dual-responsive bending and adhesive properties [138].

## 4. Conclusions

The multifunctionality of clay nanoparticles, combined with the dynamic and structural characteristics of hydrogels has proved to be useful in different fields of medicine. This combination generates synergistic effects between different materials and engineers nanocomposites with broad spectra of functionalities. The presence of clay particles in nanocomposites provided and enhanced the capacity for structural support necessary for the regeneration of new tissues by improving their load-bearing ability, slowing their degradation, and controlling the microstructure and network porosity that influence cell migration and the diffusion of oxygen and nutrients. The flow behaviors of stimuli-responsive hydrogels were tuned to successfully obtain new injectable precursor solutions for minimally invasive therapies that can jellify into mechanically robust and adhesive hydrogels. It also allowed for the production of printable bioinks with optimal filament formation and gelation temperatures. The optimizations by clay incorporation can also lead to high shape-fidelity 3D-printed constructs with physiologically bioactive characteristics. Furthermore, the binding and sustained release of molecules by the inherent mechanisms of clays, in addition to the capacity to swell and retain specific molecules of hydrogels, allows these nanocomposites to be ideal candidates for the targeted delivery of therapeutic agents. The developed scaffolds also promoted dynamic cell-scaffold interactions which are vital for superior microenvironment-integrative regeneration. Furthermore, the potential of these nanocomposites to support the regeneration of new tissues and stimulate bone growth due to gradual release of clay’s intrinsic ions, produce superior microfabricated structures with anatomical characteristics and properties common to living tissues, and function as targeted drug delivery systems capable of holding and maintaining the sustained release of different therapeutic agents with significant results, were found, among other beneficial biomedical applications. 

Since clay-based nanocomposite hydrogels demonstrate superior physical, chemical, and biological properties, their use represents a considerable advancement for biomedical science. In the future, researchers should focus on and shift their attention towards improving structural integrity, tunning the appropriate release of therapeutic agents within the desired time frame and at an effective rate, risk assessment, awareness of the effects imposed by these engineered systems within biological systems, and biodegradation. Further studies are required to better understand their mechanisms of action, the interactions between clay nanoparticles and hydrogel polymeric chains, the interactions between cells and the nanocomposites, as well as the possibility of toxic and adverse effects which a few studies demonstrated. Further developments might lead to 3D tissue models capable of providing cell support and a biomimetic environment to act as frameworks for toxicological studies. Additionally, since the fate of clay nanoparticles released from nanocomposites after their degradation is still underexplored, advanced techniques should be used to obtain insight into the physiological pathways of the released clay minerals in the body.

## Figures and Tables

**Table 1:** Clay nanoparticles in hydrogel nanocomposites for soft tissue engineering applications.

Hydrogel	Clay	Features/Observation	Ref
PEG	Laponite	Tissue adhesive hydrogel; Subcutaneous implantation in rats with minimal inflammatory response; Laponite addition promoted bioactivity, enhanced cell infiltration and adhesive performance compared to control.	[78]
GelMA + MkCA	Laponite	Gradient polymer scaffold reinforced with clay nanosilicates; Mimic native bone-cartilage interfaces; Laponite addition improved control over the mechanical, structural, and biological properties.	[66]
Gellan gum + Glycerol	HNT	HNT addition decreased water uptake and improved mechanical properties of the hydrogel scaffolds; Loading 25% of HNT induced higher metabolic activity and human fibroblast cell survival during 7 days of incubation.	[79]
PEG	LDH	Bioactive PEG hydrogel crosslinked with LDH coated with polydopamine; Nanocomposites showed self-healing ability, tunable mechanical properties, bio-adhesion; Osteogenic differentiation support of hMSCs.	[61]
PEGDA	Laponite	Laponite incorporation increased compressive and tensile properties; Not cytotoxic and supported 2D and 3D cell cultures.	[80]
PEO	Laponite	Silicate incorporation improved hMSCs’ attachment, spreading, and proliferation.	[75]
SA	HNTs	HNT incorporation improved nanocomposite’s mechanical properties, cell adhesion, and proliferation in preosteoblast (MC3T3-E1) culture.	[60]

PEG—poly (ethylene glycol); GelMa—gelatin methacryloyl; MkCA—methacrylated kappa carrageenan; HNT—halloysite nanotubes; LDH—layered double hydroxides; PEGDA—poly (ethylene glycol) diacrylate; PEO—poly (ethylene oxide); SA—sodium alginate; hMSCs—human mesenchymal stem cells.

**Table 6:** Clay nanoparticles in hydrogel nanocomposites used for bioinks and 3D printing.

Hydrogel	Clay	Features/Observation	Ref
Poly(2-methyl-2-oxazoline)-b-poly(2-n-propyl-2-oxazine))	Laponite	Thermoresponsive hybrid hydrogel exhibited high suitability for extrusion-based 3D printing and structure shape fidelity; Laponite addition retained the thermo-gelling properties, enhanced viscoelastic properties such as increased shear thinning character, and enabled a very rapid viscosity and structure recovery.	[56]
GeIMA	Laponite	High laponite concentration significantly improved the hydrogel properties and widened the fabrication window. The nanocomposite hydrogel exhibited improved rheological behaviors, mechanical strength, and stability as well as desirable printability, excellent shape fidelity, and biocompatibility. It also significantly promoted BMSC proliferation, showed high cell viability, proliferation, and osteogenic differentiation.	[76]
κCA	Laponite	The enhanced physical interaction between kCA and nanosilicates allow the printability of complex physiologically relevant tissues due to improved mechanical strength, structural integrity, and high shape fidelity of the printed filament.	[63]
Alginate, Methylcellulose	Laponite	Incorporation of laponite nanoparticles improved printability, increased shape fidelity, and conveyed a sustainable release profile of proteins (BSA and VEGF) and other biologically active agents.	[132]
Agarose	Laponite	Incorporation of laponite clay caused changes to flow behavior, elastic moduli, and gelation temperature and contributed to the formation of a highly printable hydrogel system which can retain its shape after extrusion through a fine nozzle due to enhanced structural integrity. It also significantly improved the bioactivity of nanocomposite hydrogels by means of the increased metabolic activity of encapsulated cells and the ability of cells to extend and change their morphology.	[55]
Alginate	Laponite	Laponite and alginate concentrations had significant impacts on the bioink’s overall rheological behavior (shear-thinning characteristic, viscosity profiles, flow points, filament formation, and ink printability).	[64]
HA + PEGDA	Laponite	A two-channel 3D-bioprinting method successfully fabricated an osteoblast-laden nanocomposite hydrogel construct. Bioink A (a cell-laden PEG–clay construct), not only facilitated 3D-bioprinting, but also encapsulated osteoblasts with more than 95% viability and exhibited excellent osteogenic ability, due to bioactive ion release. HA with encapsulated ROBs is applied as bioink B with a view to improving cell viability, distribution uniformity, and deposition efficiency.	[82]

GelMA—gelatine methacrylate; κCA—kappa-carrageenan; BMSC—bone marrow mesenchymal stem cell; HA—hyaluronic acid sodium salt; PEGDA—polyethylene glycol diacrylate; ROBs—primary rat osteoblasts.

## Data Availability

Not applicable.
